# Interaction between *FTO* gene variants and lifestyle factors on metabolic traits in an Asian Indian population

**DOI:** 10.1186/s12986-016-0098-6

**Published:** 2016-06-03

**Authors:** Karani S. Vimaleswaran, Dhanasekaran Bodhini, N. Lakshmipriya, K. Ramya, R. Mohan Anjana, Vasudevan Sudha, Julie A. Lovegrove, Sanjay Kinra, Viswanathan Mohan, Venkatesan Radha

**Affiliations:** Hugh Sinclair Unit of Human Nutrition and Institute for Cardiovascular and Metabolic Research (ICMR), Department of Food and Nutritional Sciences, University of Reading, Reading, UK; Department of Molecular Genetics, Madras Diabetes Research Foundation, Chennai, India; Department of Foods, Nutrition and Dietetics Research, Madras Diabetes Research Foundation, Chennai, India; Dr. Mohan’s Diabetes Specialties Centre, WHO Collaborating Centre for Non-communicable Diseases Prevention and Control, Chennai, India; Department of Non-Communicable Disease Epidemiology, London School of Hygiene & Tropical Medicine, London, UK

**Keywords:** *FTO* gene variant, Asian Indian, BMI, Obesity, Type 2 diabetes, Carbohydrate intake, Dietary fibre, Physical activity, CURES

## Abstract

**Background:**

Lifestyle factors such as diet and physical activity have been shown to modify the association between fat mass and obesity–associated (*FTO*) gene variants and metabolic traits in several populations; however, there are no gene-lifestyle interaction studies, to date, among Asian Indians living in India. In this study, we examined whether dietary factors and physical activity modified the association between two *FTO* single nucleotide polymorphisms (rs8050136 and rs11076023) (SNPs) and obesity traits and type 2 diabetes (T2D).

**Methods:**

The study included 734 unrelated T2D and 884 normal glucose-tolerant (NGT) participants randomly selected from the urban component of the Chennai Urban Rural Epidemiology Study (CURES). Dietary intakes were assessed using a validated interviewer administered semi-quantitative food frequency questionnaire (FFQ). Physical activity was based upon the self-report. Interaction analyses were performed by including the interaction terms in the linear/logistic regression model.

**Results:**

There was a significant interaction between SNP rs8050136 and carbohydrate intake (% energy) (P_interaction_ = 0.04), where the ‘A’ allele carriers had 2.46 times increased risk of obesity than those with ‘CC’ genotype (*P* = 3.0 × 10^−5^) among individuals in the highest tertile of carbohydrate intake (% energy, 71 %). A significant interaction was also observed between SNP rs11076023 and dietary fibre intake (P_interaction_ = 0.0008), where individuals with AA genotype who are in the 3^rd^ tertile of dietary fibre intake had 1.62 cm lower waist circumference than those with ‘T’ allele carriers (*P* = 0.02). Furthermore, among those who were physically inactive, the ‘A’ allele carriers of the SNP rs8050136 had 1.89 times increased risk of obesity than those with ‘CC’ genotype (P = 4.0 × 10^−5^).

**Conclusions:**

This is the first study to provide evidence for a gene-diet and gene-physical activity interaction on obesity and T2D in an Asian Indian population. Our findings suggest that the association between *FTO* SNPs and obesity might be influenced by carbohydrate and dietary fibre intake and physical inactivity. Further understanding of how *FTO* gene influences obesity and T2D through dietary and exercise interventions is warranted to advance the development of behavioral intervention and personalised lifestyle strategies, which could reduce the risk of metabolic diseases in this Asian Indian population.

**Electronic supplementary material:**

The online version of this article (doi:10.1186/s12986-016-0098-6) contains supplementary material, which is available to authorized users.

## Background

Metabolic traits such as obesity and type 2 diabetes (T2D) are generally caused by overconsumption of energy, sedentary lifestyle and genetic susceptibility [1]. Candidate gene and genome-wide association (GWA) studies have identified several common genetic variants associated with obesity and diabetes [1–5]. Of these, the fat mass and obesity–associated gene (*FTO*) variants were found to be consistently associated with obesity-related traits in several populations and has been the strongest common genetic predictor of obesity known so far [1–3, 6–9]. To date, *FTO* has shown the strongest association with BMI, where the *FTO* SNPs increased the risk of obesity by 1.20–1.32 fold in Europeans [10] and by 1.25 fold in Asians [11]. A recent meta-analysis of data from eight Indian studies showed that *FTO* variant, rs9939609, increased the risk of obesity by 1.15 times, which is equivalent to BMI increase by 0.30 kg/m^2^ per effect allele [6]. Previous study in the South Indian population (CURES) has shown that the *FTO* SNPs (rs1588413 and rs8050136) increased obesity risk by 1.27 and 2.06 times, which is equivalent to BMI increase by 0.33 and 0.54 kg/m^2^ per effect allele, respectively [7]. The *FTO* gene is abundantly expressed in hypothalamus and has been shown to be involved in the control of satiety/appetite [12]. Given its role in T2D and obesity [11, 13–16], *FTO* was considered as a candidate for our gene-diet/-physical activity interaction study. Several studies have examined the influence of lifestyle factors such as diet and physical activity on the association between *FTO* variants and obesity traits [1, 9, 15, 17]; however, there are no studies to date among Asian Indians living in India.

India has the second largest number of people with T2D in the world, where 62.4 million people in India have T2D according to the Indian Council of Medical Research-INdia DIABetes (ICMR-INDIAB) Study [18]. Asian Indians have unique clinical and biochemical characteristics that are collectively referred to as the "South Asian" or "Asian Indian Phenotype" (higher waist circumference, higher levels of total and visceral fat, hyperinsulinemia, insulin resistance, and a greater predisposition to diabetes) [19] which confers an increased susceptibility to diabetes and premature cardiovascular disease. Although there is a strong genetic component [20–25], unhealthy diet and physical inactivity have also been shown to contribute to the increasing prevalence of metabolic diseases among Asian Indians [26, 27].

Studies in European populations have shown that physical activity and dietary intake may modify the association of the *FTO* variant with obesity-related traits [28, 29]. Increased physical activity levels have been shown to attenuate the effect of *FTO* variant on obesity traits in several populations [9, 17, 28]; however, *FTO*-diet interactions have shown conflicting results, where some studies found that high energy and fat intakes or low carbohydrate intake might strengthen the association of *FTO* variants with obesity [30, 31], while others failed to confirm such interactions [14]. These inconsistencies could be attributed to differences in the sample size, genetic heterogeneity and dietary assessments. In the present study, we examined whether dietary intake and physical activity levels modified the association of the two commonly studied *FTO* single nucleotide polymorphisms (SNPs) with obesity-related traits and T2D in 1,618 individuals in the Chennai Urban Rural Epidemiology Study (CURES).

## Methods

### Study participants

CURES: The study participants were recruited from the urban component of the Chennai Urban Rural Epidemiology Study (CURES). CURES is a cross-sectional epidemiological study conducted on a representative sample of the population of Chennai (formerly Madras city) in Southern India [32]. The methodology of the study has been published elsewhere [32]. Briefly, in phase 1, 26,001 individuals were recruited based on a systematic random sampling technique. Participants with self-reported diabetes taking drug treatment for diabetes were classified as “known diabetes”. All individuals with known diabetes (*n* = 1529) were invited to visit the center for detailed studies. In addition, every 10th individual of the 26,001 individuals without known diabetes was invited to undergo oral glucose tolerance tests using a 75-g oral glucose load (dissolved in 250 ml of water) (Phase 3 of CURES). Those who were confirmed by oral glucose tolerance test to have 2-h plasma glucose value ≥ 11.1 mmol/l (200 mg/dl) based on World Health Organization (WHO) consulting group criteria were labelled as “newly detected diabetes” and those with 2-h plasma glucose value < 7.8 mmol/l (140 mg/dl) as being normal glucose tolerant (NGT) [33]. Informed consent was obtained from all study participants, and the study was approved by the Madras Diabetes Research Foundation Institutional Ethics Committee.

### Phenotype measurements

Anthropometric measurements including weight, height, and waist were obtained using standardized techniques. The BMI was calculated as weight (in kg) divided by the square of height (in m). Biochemical analyses were done on a Hitachi-912 Auto Analyzer (Hitachi, Mannheim, Germany) using kits supplied by Roche Diagnostics (Mannheim). Fasting plasma glucose (glucose oxidase–peroxidase method), serum cholesterol (cholesterol oxidase-phenol-4-amino-antipyrene peroxidase method), serum triglycerides (glycerol phosphatase oxidase-phenol-4-amino-antipyrene peroxidase method), and high-density lipoprotein cholesterol (direct method; polyethylene glycol-pretreated enzymes) were measured. Low-density lipoprotein cholesterol was calculated using the Friedewald formula [34]. Glycated haemoglobin (HbA1c) was estimated by high-performance liquid chromatography using a Variant™ machine (Bio-Rad, Hercules, CA, USA). Serum insulin concentration was estimated using an enzyme-linked immunosorbent assay (Dako, Glostrup, Denmark).

### Dietary assessment

Dietary intakes were assessed using a previously validated and published [35] interviewer administered semi-quantitative food frequency questionnaire (FFQ) containing 222 food items to estimate food intake over the past year and took between 20 and 30 min to complete. The nutritionists responsible for the data collection were well trained in the methodology to be used before the field work started. Individuals were asked to estimate the usual frequency (number of times per day, week, month or year/never) and the usual serving size of the portion of the various food items in the FFQ. Common household measures such as household cups, bowls, ladles, spoons (for the cooked foods like vegetables) wedges, circles of different diameter and visual atlas of different sizes of fruits (small, medium, large) were shown to assist the individuals in estimating portions. Information on total energy intake and macronutrients were obtained using the same FFQ. A detailed description of the development of FFQ and the data on reproducibility and validity had been published [35]. EpiNu, an in-house database was used to assess the average daily food and nutrient intake including dietary fibre intake.

Dietary glycemic load of the individual serving was calculated by multiplying the carbohydrate content (grams per serving) of each food by its glycemic index value. This glycemic load value was then multiplied by the frequency of consumption and these products were summed to obtain average daily dietary glycemic load [36].

Physical activity measurement: Physical activity was based upon a previously validated self-report questionnaire [37]. Based on exercise, leisure time activities and job related activities respondents were categorized into three groups indicating activity level (vigorously active, moderately active and sedentary). Individuals were graded as vigorously active if they did leisure time exercise and had physically demanding work, whereas individuals who either exercised or had physically demanding work were categorized as moderately active. All others were categorized as sedentary.

### SNP selection and Genotyping

The two commonly studied *FTO* SNPs (rs8050136 and rs11076023) were chosen based on their previous association with obesity and T2D in the European and South Asian populations [7, 10, 14, 17, 38]. The SNP rs8050136 lies in intron 1 of the *FTO* gene which is highly conserved across species and is located in the putative transcriptional factor (Cutl- like 1, CUTL1) binding site [39]. The rs11076023 SNP is located in the 3′UTR region of the *FTO* gene and hence it can be speculated that it might have an effect on the stability of mRNA and gene expression. The SNPs were genotyped by polymerase chain reaction on a GeneAmp PCR system 9700 thermal cycler (Applied Biosystems, Foster City, CA). The PCR products were digested with MluCI (rs8050136) and HinfI (rs11076023) restriction enzymes (New England Biolabs, Inc., Beverly, MA) and the digested products were resolved by a 3 % agarose gel electrophoresis. Based on the analysis of 200 blind duplicates (20 %), there was 100 % concordance in the genotyping. Furthermore, a few variants were confirmed by direct sequencing with an ABI 310 genetic analyzer (Foster City, CA). The *FTO* SNPs were in Hardy Weinberg equilibrium (*P* > 0.05).

### Statistical analyses

Results from the descriptive analyses are presented as means and SD for continuous variables and as percentages for categorical variables. Generalized obesity was defined according to the World Health Organization Asia Pacific Guidelines for Asians as non-obese (BMI < 25 kg/m^2^) and obese (BMI ≥ 25 kg/m^2^) [40]. A goodness-of-fit chi-square test was performed to examine if the observed genotype counts were in Hardy-Weinberg equilibrium. Student *t* test as appropriate was used to compare groups for continuous variables. Given the low frequency of the rare homozygotes, dominant model was used (comparing common homozygotes with the combined group of rare homozygotes and heterozygotes). The genetic associations with the continuous and categorical outcomes were examined using linear and logistic regression models, respectively, adjusting for age and gender. When obesity/BMI was an outcome, T2D status was adjusted and when T2D was an outcome, obesity was adjusted in the model, in addition to age and gender. Participants were divided into tertiles of carbohydrate intake (% energy) (Means: first tertile, 58 %; second tertile, 65 %; third tertile, 71 %), glycemic load (Means: first tertile, 188; second tertile, 229; third tertile, 272) and dietary fibres (Means: first tertile, 21 g/d; second tertile, 31 g/d; third tertile, 44 g/d). Interactions between each SNP and dietary intake/physical activity were assessed using linear and logistic regressions and the likelihood ratio test. The interplay between *FTO* variants and dietary intake/physical activity was investigated by including an interaction term in the regression models. All analyses were carried out using STATA, version 13. A *P* value of less than 0.05 was considered to be statistically significant.

## Results

In the CURES population, 42 % of the individuals were overweight/obese (BMI > 25 kg/m^2^). As shown in Table 1. Participants with T2D had significantly higher BMI (*P* < 0.0001), waist circumference (*P* < 0.001), systolic and diastolic blood pressures (*P* < 0.0001), fasting plasma glucose and insulin levels (*P* < 0.0001), total cholesterol (*P* < 0.0001), triglycerides (*P* < 0.0001), high-density (*P* = 0.001) and low-density (*P* < 0.0001) lipoprotein cholesterols and HbA1c (*P* < 0.0001). *FTO* gene variants, rs8050136 and rs11076023, have been shown to be associated with obesity and T2D, respectively, in the CURES study [7].

**Table 1:** Clinical and biochemical characteristics of the participants from the CURES study

Clinical and biochemical parameters	Type 2 diabetic subjects (T2DM)		Normal glucose tolerance subjects (NGT)		*P* value*
	Men	Women	Men	Women	
	(*N* = 407)	(*N* = 327)	(*N* = 509)	(*N* = 375)	
Age (yrs)	49.59 ± 10.49	50.74 ± 11.47	36.25 ± 11.21	36.54 ± 11.70	<0.0001
BMI (Kg/m^2^)	26.33 ± 4.37	24.49 ± 3.94	23.57 ± 5.09	22.74 ± 4.25	<0.0001
WC (cm)	90.08 ± 9.95	92.11 ± 9.93	80.76 ± 12.43	83.81 ± 11.85	<0.0001
Obese cases (%)	243.00 (60.00)	139.00 (42.50)	179.00 (35.20)	110.00 (29.40)	<0.0001
Fasting plasma glucose (mg/dl)	165.42 ± 73.22	155.71 ± 61.52	84.47 ± 8.36	84.71 ± 8.14	<0.0001
Fasting serum insulin (μIU/ml)	12.68 ± 7.62	10.61 ± 6.95	8.48 ± 5.98	7.48 ± 4.88	<0.0001
Systolic blood pressure (mmHg)	129.91 ± 21.82	128.11 ± 21.72	114.25 ± 17.54	116.25 ± 15.60	<0.0001
Diastolic blood pressure (mmHg)	76.54 ± 12.07	78.03 ± 11.73	71.25 ± 11.38	74.28 ± 11.52	<0.0001
Total serum cholesterol (mg/dl)	209.21 ± 43.71	193.66 ± 39.34	173.35 ± 35.15	171.99 ± 35.91	<0.0001
Fasting serum triglycerides (mg/dl)	179.10 ± 118.48	189.25 ± 145.81	100.88 ± 53.71	123.67 ± 70.97	<0.0001
HDL cholesterol (mg/dl)	43.68 ± 9.76	39.05 ± 8.66	45.47 ± 9.80	40.10 ± 9.49	<0.0001
LDL cholesterol (mg/dl)	131.76 ± 36.19	120.93 ± 33.66	107.90 ± 30.17	107.47 ± 29.78	<0.0001
Glycated Haemoglobin (HbA1c)	8.65 ± 2.39	8.76 ± 2.32	5.54 ± 0.50	5.55 ± 0.49	<0.0001
PAL [Sedentary (%); Moderate (%); Vigorous (%)]	263 (77.10); 76 (22.30); 2 (0.60)	193 (46.80); 60 (20.10); 45 (15.10)	362 (80.80); 80 (17.90); 6 (1.30)	240 (64.70); 112 (30.20); 19 (5.10)	0.001
Total Carbohydrate energy %	65.50 ± 5.43	64.08 ± 6.27	64.60 ± 5.89	63.09 ± 6.84	0.003
Fat energy %	23.23 ± 4.51	23.58 ± 4.76	23.81 ± 4.79	23.88 ± 4.65	0.05
Protein energy %	11.47 ± 1.20	11.43 ± 1.25	11.25 ± 1.12	11.35 ± 1.25	0.009
Energy adjusted glycemic index	62.76 ± 0.14	62.16 ± 0.18	62.94 ± 0.12	62.65 ± 0.14	0.02
Energy adjusted glycemic load	233.52 ± 1.64	226.12 ± 2.40	231.63 ± 1.74	224.32 ± 0.36	0.41
Dietary fibre (g)	28.62 ± 0.54	34.91 ± 0.69	29.84 ± 0.42	35.73 ± 0.56	0.10

NGT, Normal Glucose Tolerance; BMI, Body Mass Index; WC, Waist Circumference; HDL, High Density Lipoproteins; LDL, Low Density Lipoproteins; PAL, Physical Activity Level

Data shown are represented as means ± SD, wherever appropriate

*P values for the differences in the means/proportions between Type 2 diabetes cases and NGT participants

### Interactions between *FTO* variants and dietary intake on obesity traits and T2D

**Fig. 1:**
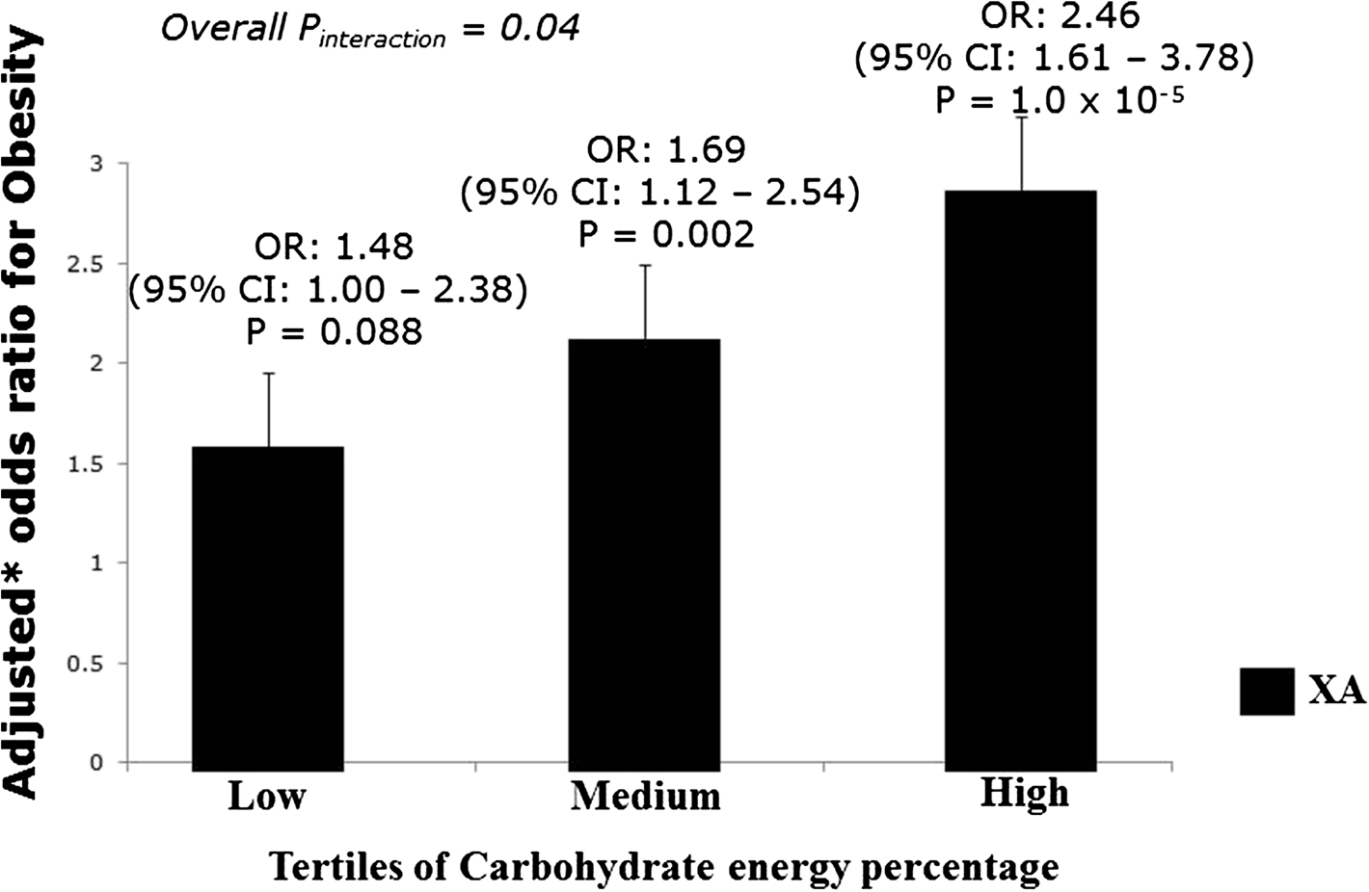
Interaction of the *FTO* gene polymorphism (rs8050136) with carbohydrate energy percentage on obesity. The ‘A’ allele carriers (XA = CA + AA) who are in the 3^rd^ tertile of carbohydrate energy percentage have 2.46 times increased risk of obesity (*P* = 1.0 × 10^−5^). *Odds ratio adjusted for age, gender and diabetes

There was a significant interaction between *FTO* SNP rs8050136 and carbohydrate intake % energy) (P_interaction_ = 0.04) on obesity, where the ‘A’ allele carriers of the SNP rs8050136 had 2.46 times increased risk of obesity than those with ‘CC’ genotype (*P* = 1.0 × 10^−5^) among individuals in the highest tertile of carbohydrate intake (% energy, 71 %) (Fig. 1). We also found a borderline interaction between SNP rs8050136 and energy adjusted glycemic load (P_interaction_ = 0.07) on obesity (Additional file 1: Figure S1), where the ‘A’ allele carriers of the SNP rs8050136 had 2.31 times increased risk of obesity than those with ‘CC’ genotype among individuals in the highest tertile of energy adjusted glycemic load (*P* = 6.0 × 10^−5^).

**Fig. 2:**
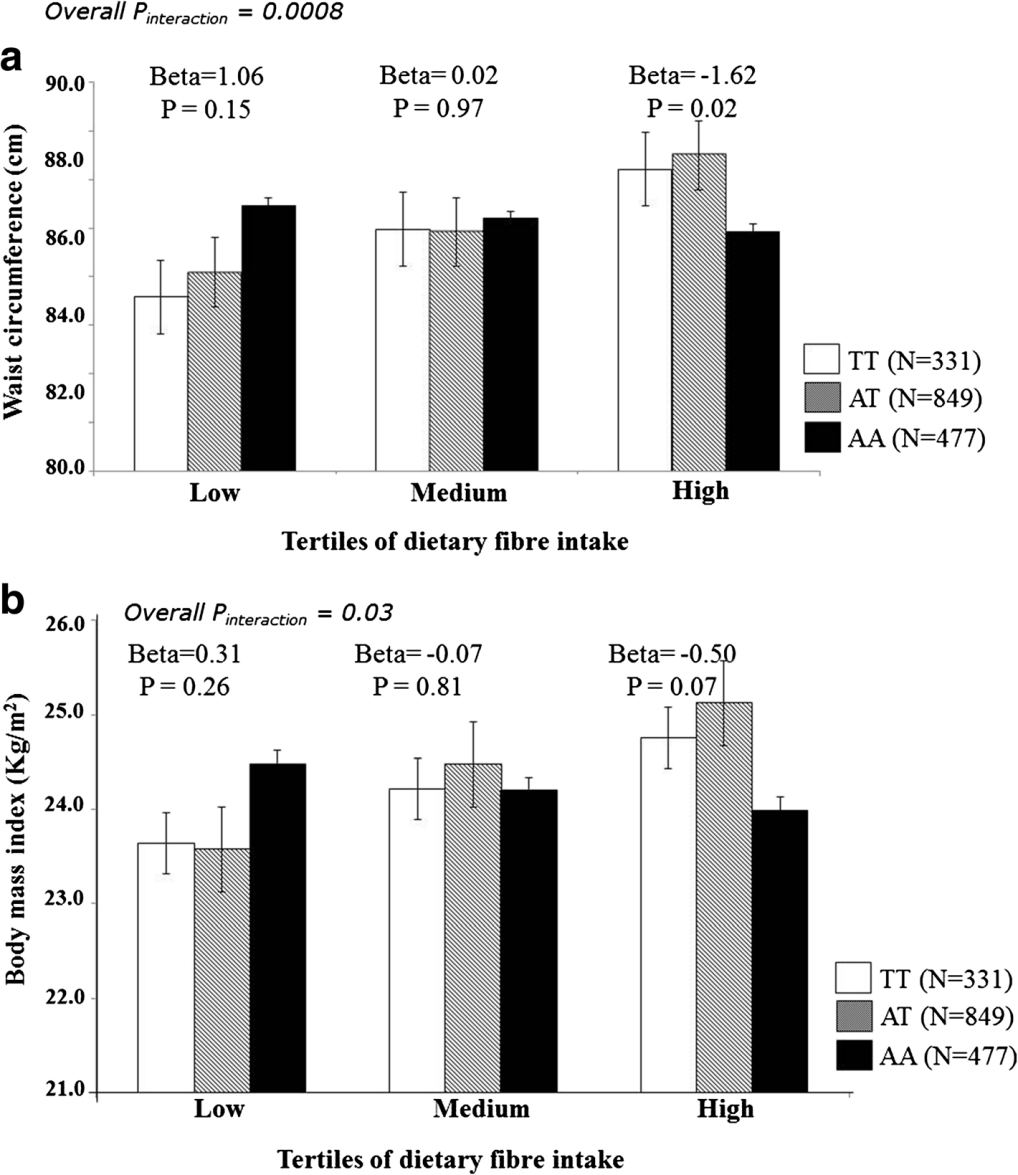
**a** Interaction of the *FTO* gene polymorphism (rs11076023) with dietary fibre intake on waist circumference. The individuals with AA genotype who are in the 3^rd^ tertile of dietary fibre intake have 1.62 cm decrease in waist circumference compared to those with ‘T’ allele carriers (*P* = 0.02). **b** Interaction of the *FTO* gene polymorphism (rs11076023) with dietary fibre intake on body mass index. The individuals with AA genotype who are in the 3^rd^ tertile of dietary fibre intake have 0.50 kg/m^2^ decrease in body mass index compared to those with ‘T’ allele carriers (*P* = 0.07)

With the SNP rs11076023, we observed a significant interaction (P_interaction_ = 0.0008), where individuals with ‘AA’ genotype who are in the 3^rd^ tertile of dietary fibre intake (44 g/d) had 1.62 cm decrease in waist circumference than those with ‘T’ allele carriers (*P* = 0.02) (Fig. 2a). A similar interaction trend, but less stronger, was observed with BMI (P_interaction_ = 0.03), where individuals with ‘AA’ genotype who are in the 3^rd^ tertile of dietary fibre intake (44 g/d) had 0.50 kg/m^2^ decrease in BMI than those with ‘T’ allele carriers (*P* = 0.07) (Fig. 2b). A borderline interaction (P_interaction_ = 0.09) was also seen, where the ‘A’ allele carriers of the SNP rs11076023 in the highest tertile of carbohydrate (% energy, mean: 71 %) had 1.57 times increased risk of T2D than those with ‘TT’ genotype (*P* = 0.002) (Additional file 1: Figure S2), while the first (mean: 58 %) and second (mean: 65 %) tertiles did not show any evidence for an association of the SNP rs11076023 with T2D.

None of the other interactions between the *FTO* SNPs and fat and protein energy percentages, glycemic index and dietary fibre on obesity traits and T2D were statistically significant (P_interaction_ > 0.05 for all interactions) (Tables 2 and 3).

**Table 2:** Interaction of the *FTO* SNP (rs8050136) with dietary factors on BMI, obesity, waist circumference and Type 2 diabetes

SNP * Fat energy percentage	SNP * Protein energy percentage	SNP * Carbohydrate energy percentage	SNP * energy adjusted glycemic load	SNP * energy adjusted glycemic index	SNP * dietary fibre
Beta coefficients ± standard error (P_interaction_)^¥^ for interaction on BMI					
−0.06 ± 0.06 (0.27)	−0.16 ± 0.23 (0.49)	0.02 ± 0.04 (0.67)	0.002 ± 0.007 (0.81)	0.05 ± 0.09 (0.60)	−0.01 ± 0.02 (0.55)
Beta coefficients ± standard error (P_interaction_)^¥^ for interaction on obesity					
−0.05 ± 0.03 (0.07)	−0.11 ± 0.11(0.29)	0.04 ± 0.02 (0.04)	0.005 ± 0.003 (0.08)	0.05 ± 0.04 (0.21)	−0.008 ± 0.01 (0.45)
Beta coefficients ± standard error (P_interaction_)^¥^ for interaction on waist circumference					
−0.14 ± 0.14 (0.34)	−0.27 ± 0.57 (0.64)	0.04 ± 0.11 (0.68)	0.004 ± 0.02 (0.83)	0.20 ± 0.23 (0.38)	−0.03 ± 0.06 (0.57)
Beta coefficients ± standard error (P_interaction_)** for interaction on Type 2 diabetes					
0.01 ± 0.03 (0.73)	−0.03 ± 0.12 (0.78)	0.007 ± 0.02 (0.73)	0.00008 ± 0.003 (0.98)	−0.02 ± 0.05 (0.70)	0.03 ± 0.01 (0.01)

^¥^ *P* values adjusted for age, gender and type 2 diabetes

**P values adjusted for age, gender and obesity

**Table 3:** Interaction of the *FTO* SNP (rs11076023) with dietary factors on BMI, obesity, waist circumference and Type 2 diabetes

SNP * Fat energy percentage	SNP * Protein energy percentage	SNP * Carbohydrate energy percentage	SNP * energy adjusted glycemic load	SNP * energy adjusted glycemic index	SNP * dietary fibre
Beta coefficients ± standard error (P_interaction_)^¥^ for interaction on BMI					
0.009 ± 0.04 (0.79)	0.10 ± 0.14 (0.44)	0.004 ± 0.03 (0.89)	0.003 ± 0.004 (0.52)	0.03 ± 0.06 (0.57)	−0.03 ± 0.01 (0.03)
Beta coefficients ± standard error (P_interaction_)^¥^ for interaction on obesity					
0.0004 ± 0.01 (0.98)	−0.04 ± 0.06 (0.50)	0.004 ± 0.01 (0.74)	0.001 ± 0.002 (0.59)	0.01 ± 0.03 (0.59)	−0.01 ± 0.007 (0.17)
Beta coefficients ± standard error (P_interaction_)^¥^ for interaction on waist circumference					
−0.12 ± 0.0.09 (0.18)	−0.32 ± 0.34 (0.34)	0.11 ± 0.07 (0.12)	0.02 ± 0.01 (0.04)	0.25 ± 0.14 (0.08)	−0.12 ± 0.04 (0.0008)
Beta coefficients ± standard error (P_interaction_)** for interaction on Type 2 diabetes					
−0.03 ± 0.02 (0.15)	−0.08 ± 0.07 (0.27)	0.02 ± 0.01 (0.09)	0.003 ± 0.002 (0.16)	0.02 ± 0.03 (0.53)	−0.007 ± 0.007 (0.39)

^¥^ *P* values adjusted for age, gender and type 2 diabetes

**P values adjusted for age, gender and obesity

### Interactions between *FTO* variants and physical activity levels on obesity traits and T2D

We observed interactions between the SNP rs8050136 and physical activity on BMI, waist circumference and obesity (Additional file 1: Figure S3a, b and c). Even though the overall interaction P values were not statistically significant (*P* > 0.11 for all comparisons), sub-group analysis within each levels of physical activity revealed stronger associations of the SNP rs8050136 with obesity traits among those who were physically inactive. Among those who were physically inactive, the ‘A’ allele carriers of the SNP rs8050136 had 0.95 kg/m^2^ per minor allele increase in BMI (*P* = 0.002), 3.06 cm per minor allele increase in waist circumference (*P* = 2 × 10^−4^) and 1.89 times increased risk of obesity (*P* = 4.0 × 10^−5^), compared to those with ‘CC’ genotype. Given that the overall interaction was not significant and the effect was observed only after stratification based on physical activity levels, we tested for the association between the SNP and physical activity levels to avoid spurious interaction effects due to collider bias [41]. There was no association between the SNP rs8050136 and physical activity levels (*P* = 0.53) suggesting that the observed interaction is not spurious.

We also observed a statistically significant interaction of the SNP rs8050136 with physical activity on T2D (P_interaction_ = 0.003) (Table 4). None of the other interactions with physical activity levels on obesity traits and T2D were statistically significant (P_interaction_ > 0.05 for all interactions).

**Table 4:** Interaction of the *FTO* gene polymorphisms (rs8050136 and rs11076023) with physical activity levels on BMI, obesity and Type 2 diabetes

Beta coefficients ± standard error (P_interaction_ *) for interaction on BMI	
rs8050136	−0.76 ± 0.48 (0.11)
rs11076023	−0.32 ± 0.29 (0.27)
Beta coefficients ± standard error (P_interaction_ *) for interaction on obesity	
rs8050136	−0.21 ± 0.24 (0.38)
rs11076023	0.002 ± 0.15 (0.99)
Beta coefficients ± standard error (P_interaction_ *) for interaction on waist circumference	
rs8050136	−1.86 ± 1.29 (0.14)
rs11076023	−0.29 ± 0.77 (0.70)
Beta coefficients ± standard error (P_interaction_ **) for interaction on Type 2 diabetes	
rs8050136	0.91 ± 0.32 (0.003)
rs11076023	0.24 ± 0.18 (0.18)

*P values adjusted for age, gender and type 2 diabetes

** *P* values adjusted for age, gender and obesity

## Discussion

Our study provides the first gene-diet interaction data on obesity-related traits and T2D in an Asian Indian population. The present study shows that carbohydrate and dietary fibre intake may modify the association of the *FTO* SNPs, rs8050136 and rs11076023, with obesity traits, with the effect of the SNP being more pronounced among those who consumed high levels of carbohydrate and dietary fibre. The study also provides evidence that low levels of physical activity may accentuate the risk of obesity by the *FTO* SNP rs8050136. Given the high prevalence of T2D in South Asia and as 28–44 % of Asians carry at least one copy of the *FTO* risk allele [11], our findings hold significant implications for public health.

While carbohydrates do not constitute the bulk of the energy in the West (~45 %) [42], the diet is high in carbohydrates among South Asian countries such as India (65.6 %) [36, 43]. Furthermore, higher dietary carbohydrates and glycemic load have been shown to be associated with the risk of T2D among urban south Indians [43]. In the present study, we found a significant interaction between the *FTO* SNP rs8050136 and carbohydrate energy percentage on obesity, where the minor allele carriers had higher risk of obesity among those in the highest tertile of carbohydrate energy percentage. While our findings are in contrast to the large European studies which failed to find an interaction between *FTO* and dietary carbohydrate intake on obesity [14, 44, 45], a study in 4,895 Swedish individuals [30] showed a significant interaction (P_interaction_ = 0.0004); however, the effect allele of the *FTO* SNP rs9939609 (strong LD with rs8050136) increased the risk of obesity among those with low carbohydrate and high fat intakes. In the present study, the total carbohydrate intake was much higher (first tertile, 276 g/d; third tertile, 560 g/d) compared with that reported in the Western population (first quintile, 162 g/d; fifth quintile, 238 g/d) [46] and in another Asian population (first quintile, 233.3 g/d; fifth quintile, 321.9 g/d) [47]. This might be one of the reasons why the previous European studies on obesity failed to identify an interaction with carbohydrate intake and also this finding partly explains the role of varying dietary patterns in contributing to the heterogeneity in identifying and replicating gene-diet interactions.

The positive interaction that was observed between SNP rs11076023 and dietary fibre intake on BMI and waist circumference in our study (mean dietary fibre intake, our study: ~32 g/d) is consistent with the previous report from the Finnish Diabetes Prevention Study (mean dietary fibre intake, Finnish: ~20 g/d) [48], where higher BMI was observed in those who had a diet low in dietary fibre. Furthermore, a recent study from the CURES also showed an inverse association between dietary fibre intake and cardiovascular disease (CVD) risk factors among those with T2D, where individuals who consumed dietary fibre <29 g/d had a higher levels of total and LDL cholesterol [49]. In our study, the interaction between dietary fibre intake and SNP rs11076023 was more pronounced on waist circumference than on BMI, which suggests that effects are likely to be on central obesity rather than obesity per se. This was indeed shown in another Asian (Japanese) population where dietary fibre intake was associated with reduced prevalence of abdominal obesity after multivariate adjustments including obesity in 4,399 individuals [50].

The prevalence of physical inactivity among South East Asians (17 %) has been shown to be lower than that of Europeans (34.8 %) [51]. However, a recent study in 14,227 individuals has shown that 54.4 % of the Indian population is physically inactive [26]. Significant interaction between *FTO* and physical inactivity on obesity has been reported in several studies from Europe and Asia [9, 17]; however, there are no studies, to date, among Asian Indians living in India. In the present study, we found that among those who were physically inactive, the minor allele carriers of the *FTO* SNP rs8050136 had higher risk of obesity. Our finding is in accordance with the previous large studies among Europeans [9, 17] where physical activity was shown to attenuate the risk of *FTO* risk allele on obesity.

Our study has several limitations. We performed a cross-sectional study and hence, we are unable to infer causality between the consumption of carbohydrate-rich foods/physical inactivity and development of obesity. Although confounders were adjusted in all our analyses, residual confounding due to unknown factors cannot be excluded. Interactions were significant only with total carbohydrate intake (energy %) but not when split as total and added sugars and refined and whole cereals (data not shown). Even though our study has a small sample size compared to the large European cohorts, we were still able to confirm the previously reported interactions between *FTO* variants and dietary intake and physical activity on obesity traits in this Asian Indian population. The small sample size might be one of the reasons why there was no consistency in the gene–diet interactions, where SNP rs8050136 showed an interaction with carbohydrate intake (energy %) while SNP rs11076023 showed an interaction with dietary fibre. However, we cannot rule out the fact that complex traits such as obesity are caused by multiple gene variants and lifestyle factors and hence, it is not necessary for all the variants in the gene to show interaction with the same dietary factor. Given the questionnaire based assessment of physical activity levels, measurement error cannot be completely excluded. Finally, even though we adjusted for age in all the analyses, it is possible that the unmatched age in cases and controls can introduce a bias in the study. Two main strengths of our study is the validated FFQ, which has shown high reproducibility and validity for total carbohydrates, dietary fibre, glycemic index and glycemic load, and the analysis of interactions in a well characterised population.

## Conclusions

In summary, this is the first study to provide evidence for a gene-diet and gene-physical activity interaction on obesity and T2D in an Asian Indian population. Our findings suggest that the effect of the *FTO* SNPs, rs8050136 and rs11076023, on obesity is influenced by high carbohydrate and dietary fibre intakes and low physical activity levels. Given that India leads the world in diabetes [52], our study highlights the need to consume foods with low carbohydrate content and high dietary fibre and to increase physical activity levels, as these could substantially reduce the genetic risk of obesity and diabetes. Further understanding of how *FTO* gene influences obesity and T2D through dietary and exercise interventions is warranted to advance the development of behavioral intervention and personalised lifestyle strategies, which could reduce the risk of metabolic diseases in this Asian Indian population.

## Additional file

Additional file 1:
**Figure S1:** Interaction of the *FTO* gene polymorphism (rs8050136) with energy adjusted glycemic load on obesity. The ‘A’ allele carriers had 2.31 times increased risk of obesity than those with ‘CC’ genotype among individuals in the highest tertile of energy adjusted glycemic load (P=6.0x10^-5^). Figure S2: Interaction of the *FTO* gene polymorphism (rs11076023) with carbohydrate energy percentage on Type 2 diabetes. The ‘A’ allele carriers who are in the 3^rd^ tertile of carbohydrate energy percentage have 1.66 times increased risk of Type 2 diabetes (P=0.002). *Odds ratio adjusted for age, gender and obesity. Figure S3a: Interaction of the *FTO* gene polymorphism (rs8050136) with physical activity level on body mass index (BMI). The ‘A’ allele carriers who are physically inactive have 0.95 kg/m^2^ increase in BMI compared to those with ‘C’ allele carriers (P = 0.002). Figure S3b: Interaction of the *FTO* gene polymorphism (rs8050136) with physical activity level on waist circumference. The ‘A’ allele carriers who are physically inactive have 2.90cm higher waist circumference compared to those with ‘C’ allele carriers (P = 2 × 10^-4^). Figure S3c: Interaction of the *FTO* gene polymorphism (rs8050136) with physical activity level on obesity. The ‘A’ allele carriers who are physically inactive have 1.89 times increased risk of obesity compared to those with ‘C’ allele carriers (P = 4 x 10^-5^). *Odds ratio adjusted age, gender and Type 2 diabetes. (DOCX 259 kb)

## Abbreviations

CURES: Chennai Urban Rural Epidemiological Study
*FTO*: Fat mass and obesity associated
NGT: Normal glucose tolerance
T2D: Type 2 diabetes
