# Erratum to: Interaction between FTO gene variants and lifestyle factors on metabolic traits in an Asian Indian population

**DOI:** 10.1186/s12986-016-0100-3

**Published:** 2016-06-13

**Authors:** Karani S. Vimaleswaran, Dhanasekaran Bodhini, N. Lakshmipriya, K. Ramya, R. Mohan Anjana, Vasudevan Sudha, Julie A. Lovegrove, Sanjay Kinra, Viswanathan Mohan, Venkatesan Radha

**Affiliations:** Hugh Sinclair Unit of Human Nutrition and Institute for Cardiovascular and Metabolic Research (ICMR), Department of Food and Nutritional Sciences, University of Reading, Reading, UK; Department of Molecular Genetics, Madras Diabetes Research Foundation, Chennai, India; Department of Foods, Nutrition and Dietetics Research, Madras Diabetes Research Foundation, Chennai, India; Dr. Mohan’s Diabetes Specialties Centre, WHO Collaborating Centre for Non-communicable Diseases Prevention and Control, Chennai, India; Department of Non-Communicable Disease Epidemiology, London School of Hygiene & Tropical Medicine, London, UK

## Erratum

After publication of the original article [1], the authors noticed an error in the ‘Acknowledgements’ section. “(CURES-79)” should be “(CURES-137)”. The correct ‘Acknowledgements’ section has been included in this erratum accordingly:

**Acknowledgements**

Dr Karani S Vimaleswaran acknowledges support from the British Nutrition Foundation. The study was supported by Lady Tata Memorial Trust, Mumbai. The Chennai Wellingdon Corporate Foundation supported the CURES field studies (CURES-137).
