# Supplementary material for: Interaction between FTO gene variants and lifestyle factors on metabolic traits in an Asian Indian population
Source: Nutr Metab (Lond). 2016 Jun 3;13:39. doi: 10.1186/s12986-016-0098-6 (PMC4891824; doi:10.1186/s12986-016-0098-6)

**Supplementary figure 1:** Interaction of the *FTO* gene polymorphism (rs8050136) with energy adjusted glycemic load on obesity. The ‘A’ allele carriers had 2.31 times increased risk of obesity than those with ‘CC’ genotype among individuals in the highest tertile of energy adjusted glycemic load (P=6.0x10^-5^).

**
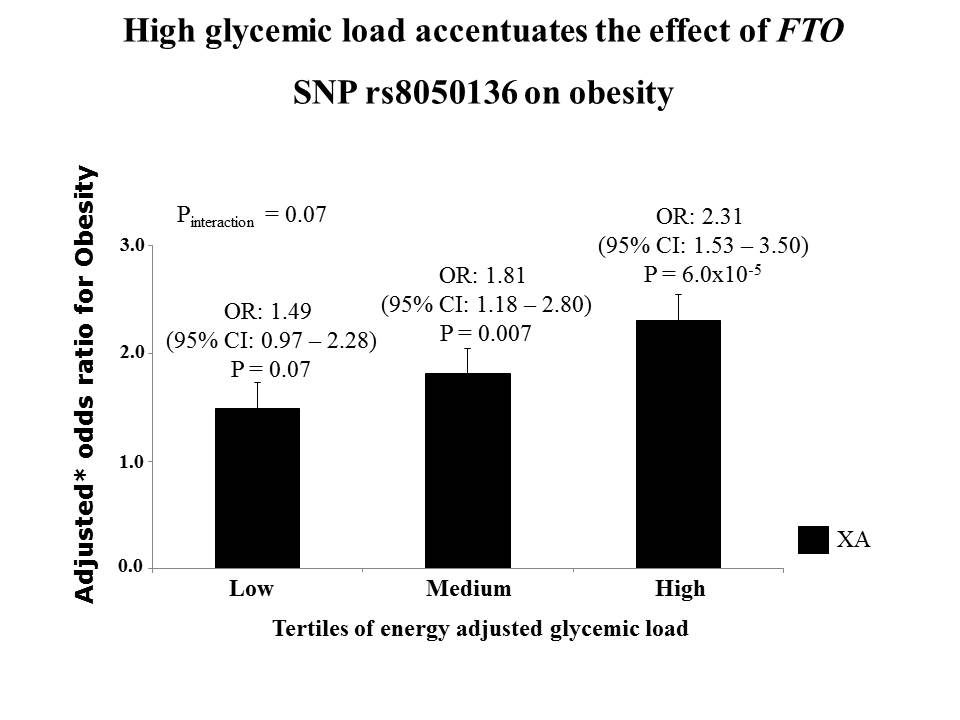
**

**Supplementary figure 2:** Interaction of the *FTO* gene polymorphism (rs11076023) with carbohydrate energy percentage on Type 2 diabetes. The ‘A’ allele carriers who are in the 3^rd^ tertile of carbohydrate energy percentage have 1.66 times increased risk of Type 2 diabetes (P=0.002). *Odds ratio adjusted for age, gender and obesity.

**
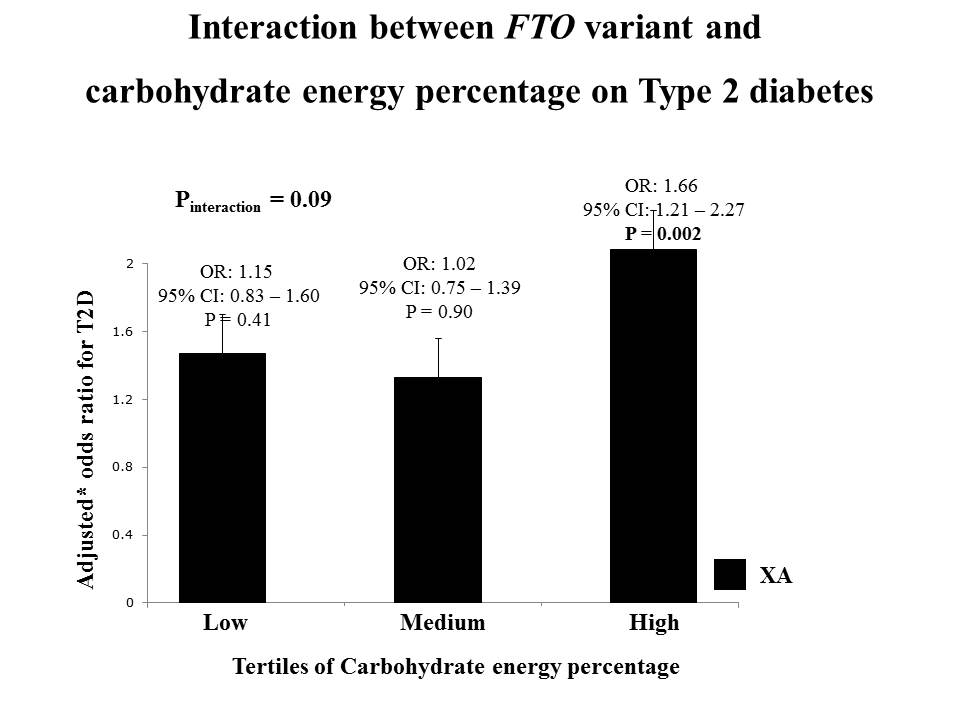
**

**Supplementary figure 3a: Interaction of the *FTO* gene polymorphism (rs8050136) with physical activity level on body mass index (BMI). The ‘A’ allele carriers who are physically inactive have 0.95 kg/m^2^ increase in BMI compared to those with ‘C’ allele carriers (P = 0.002).**


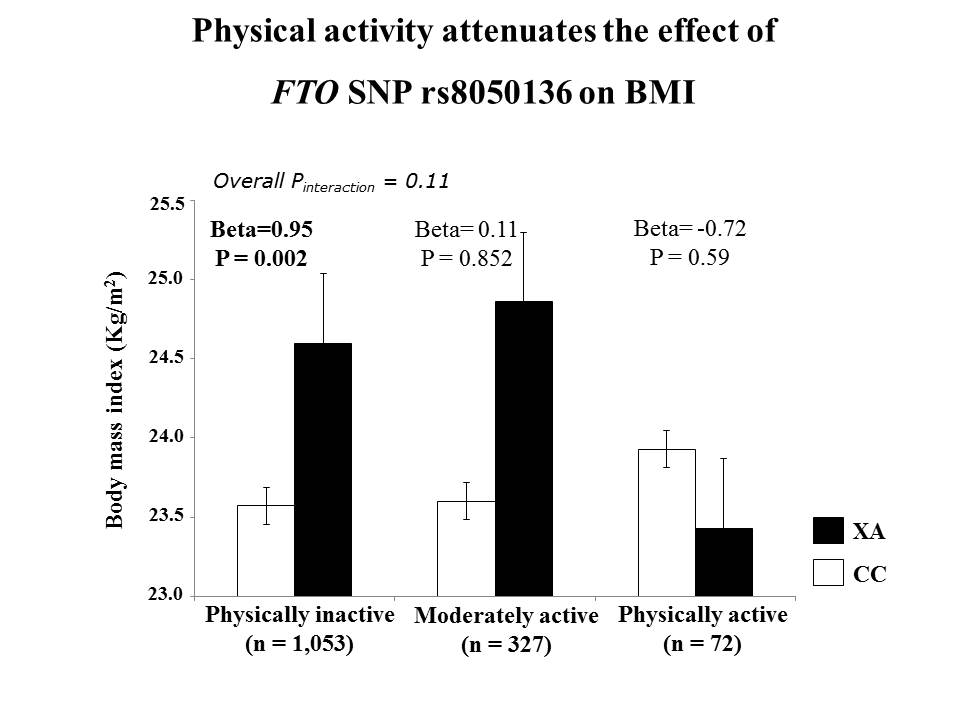


**Supplementary figure 3b: Interaction of the *FTO* gene polymorphism (rs8050136) with physical activity level on waist circumference. The ‘A’ allele carriers who are physically inactive have 2.90cm higher waist circumference compared to those with ‘C’ allele carriers (P = 2 x 10^-4^).**

**
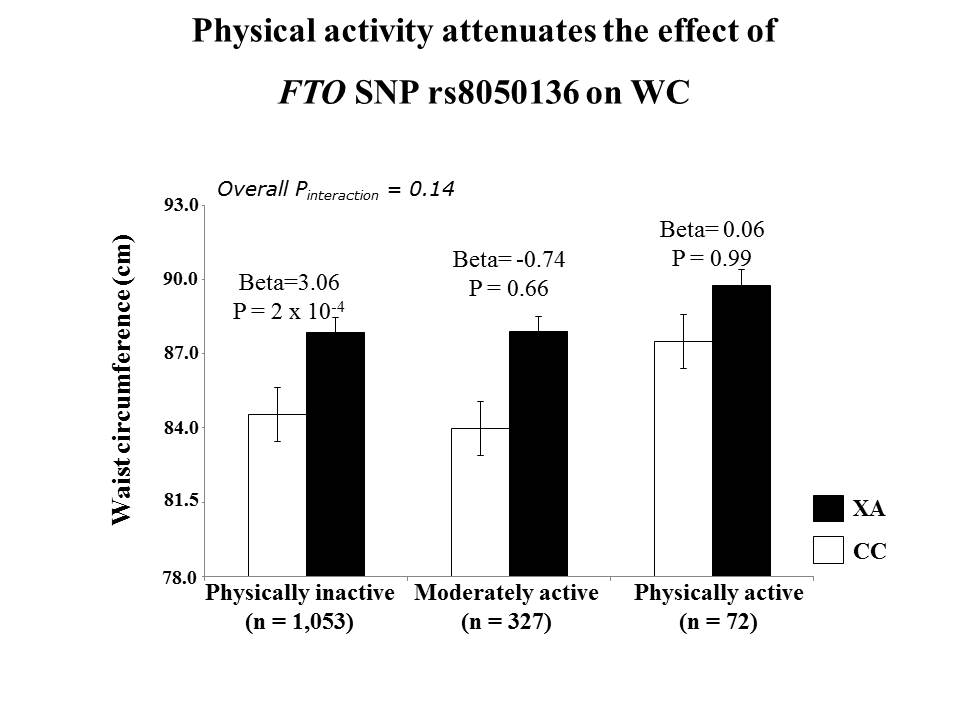
**

**Supplementary figure 3c: Interaction of the *FTO* gene polymorphism (rs8050136) with physical activity level on obesity. The ‘A’ allele carriers who are physically inactive have 1.89 times increased risk of obesity compared to those with ‘C’ allele carriers (P = 4 x 10^-5^). *Odds ratio adjusted age, gender and Type 2 diabetes.**


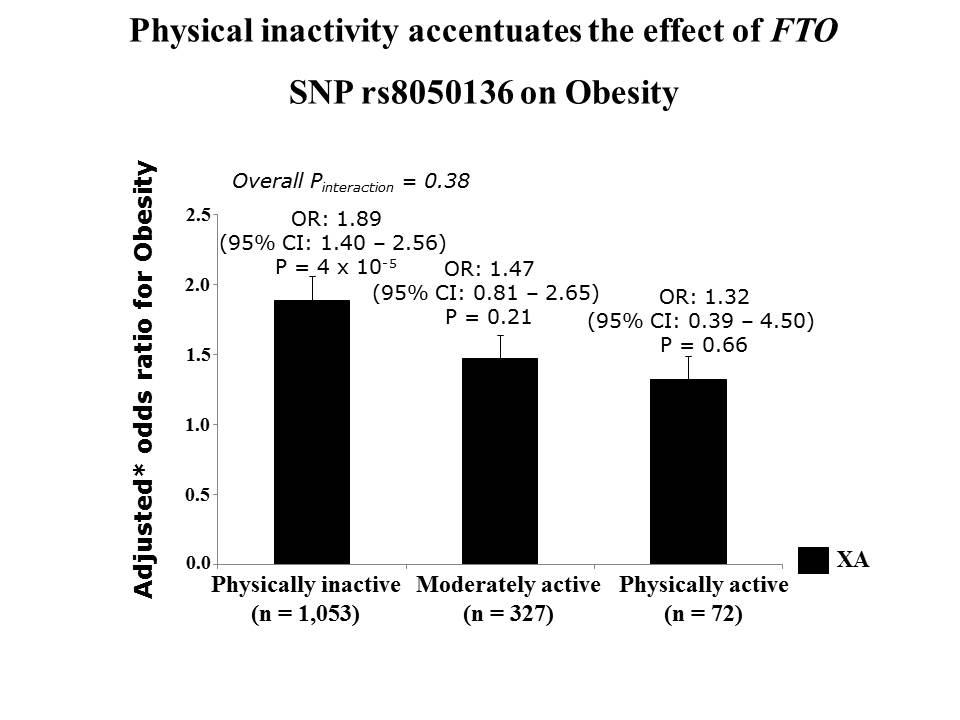

Supplement: Additional file 1: — Figure S1: Interaction of the FTO gene polymorphism (rs8050136) with energy adjusted glycemic load on obesity. The ‘A’ allele carriers had 2.31 times increased risk of obesity than those with ‘CC’ genotype among individuals in the highest tertile of energy adjusted glycemic load (P=6.0x10^-5^). Figure S2: Interaction of the FTO gene polymorphism (rs11076023) with carbohydrate energy percentage on Type 2 diabetes. The ‘A’ allele carriers who are in the 3rd tertile of carbohydrate energy percentage have 1.66 times increased risk of Type 2 diabetes (P=0.002). *Odds ratio adjusted for age, gender and obesity. Figure S3a: Interaction of the FTO gene polymorphism (rs8050136) with physical activity level on body mass index (BMI). The ‘A’ allele carriers who are physically inactive have 0.95 kg/m2 increase in BMI compared to those with ‘C’ allele carriers (P = 0.002). Figure S3b: Interaction of the FTO gene polymorphism (rs8050136) with physical activity level on waist circumference. The ‘A’ allele carriers who are physically inactive have 2.90cm higher waist circumference compared to those with ‘C’ allele carriers (P = 2 × 10^-4^). Figure S3c: Interaction of the FTO gene polymorphism (rs8050136) with physical activity level on obesity. The ‘A’ allele carriers who are physically inactive have 1.89 times increased risk of obesity compared to those with ‘C’ allele carriers (P = 4 x 10^-5^). *Odds ratio adjusted age, gender and Type 2 diabetes. (DOCX 259 kb) [file 12986_2016_98_MOESM1_ESM.docx]
